# Comparison of acute vertigo diagnosis and treatment practices between otolaryngologists and non-otolaryngologists: A multicenter scenario-based survey

**DOI:** 10.1371/journal.pone.0213196

**Published:** 2019-03-07

**Authors:** Kenji Numata, Takashi Shiga, Kazuhiro Omura, Akiko Umibe, Eiji Hiraoka, Shunsuke Yamanaka, Hiroyuki Azuma, Yasuhiro Yamada, Daiki Kobayashi

**Affiliations:** 1 Department of Emergency Medicine, Tokyo Bay Urayasu Ichikawa Medical Center, Todaijima, Urayasu city, Chiba, Japan; 2 Department of Emergency Medicine, International University of Health and Welfare, Mita hospital, Mita, Minato-ku Tokyo, Japan; 3 Department of Otorhinolaryngology, The Jikei University School of Medicine, Nishi-shimbashi, Minato-ku, Tokyo, Japan; 4 Department of Otorhinolaryngology, Dokkyo Medical University Saitama Medical Center, Minamikoshigaya, Koshigaya city, Saitama, Japan; 5 Department of General Medicine, Tokyo Bay Urayasu Ichikawa Medical Center, Todaijima, Urayasu city, Chiba, Japan; 6 Department of Emergency Medicine, University of Fukui Hospital, Matsuokashimoaizuki, Eiheiji-cho, Yoshida-gun, Fukui, Japan; 7 Department of Emergency Medicine, Fukui prefectural hospital, Fukui, Fukui prefecture, Japan; 8 Department of General Internal Medicine, National Hospital Organization Tokyo Medical Center, higashigaoka, meguro-ku, Tokyo, Japan; 9 Division of General Internal Medicine, St. Luke’s International Hospital, Tokyo Japan; University College London, UNITED KINGDOM

## Abstract

Acute vertigo is a common problem in emergency departments. However, clinical strategies of acute vertigo care vary among care providers. The aim of the study was to investigate differences in diagnosis [Dix-Hallpike test, the head impulse, nystagmus, and the test of skew (HINTS) procedure, and imaging modalities] and treatment (pharmacological treatments and the Epley maneuver) by otolaryngologists and non-otolaryngologists in emergency medicine settings. We used a multicenter case-based survey for the study. Four clinical vignettes of acute vertigo (posterior canal benign paroxysmal positional vertigo, vestibular neuritis, Meniere disease, and nonspecific vertigo) were used. Total 151 physicians from all study sites participated in the study. There were 84 non-otolaryngologists (48 emergency physicians and 36 internists) and 67 otolaryngologists. The multivariate analysis indicated that otolaryngologists ordered fewer CT scans (odds ratio (OR), 0.20; 95% confidence interval (CI), 0.07–0.53) and performed fewer HINTS procedures (OR, 0.17; 95% CI, 0.06–0.46), but used the Dix-Hallpike method more often (OR, 2.36; 95% CI, 1.01–5.52) for diagnosis compared to non-otolaryngologists. For treatment, otolaryngologists were less likely to use the Epley method (OR, 0.19; 95% CI, 0.07–0.53) and metoclopramide (OR, 0.09; 95% CI, 0.01–0.97) and more likely to use sodium bicarbonate (OR, 20.50; 95% CI, 6.85–61.40) compared to non-otolaryngologists. We found significant differences in the acute vertigo care provided by non-otolaryngologists and otolaryngologists from a vignette-based research. To improve acute vertigo care, educational systems focusing on acute vertigo are needed.

## Introduction

Acute vertigo is a common and challenging problem encountered in the departments of otolaryngology, emergency medicine (EM), and internal medicine (IM). More than one-third of Americans visit a health care provider for dizziness during their life-time [1]. Vertigo is one of the most common complaints in the emergency department (ED) and is responsible for 2.5% of ED visits [2–4]. The psychological impact of vertigo can significantly affect an individual’s lifestyle. Patients with vertigo have had to quit, change, or modify their job because of their symptoms [5]. Appropriate treatment is important to lead the patients to complete recovery [6].

However, there is variation in the guidelines regarding acute vertigo diagnosis and management in several specialties. Inconsistency in the available literature has also hindered the emergence of a consensus in the evidence used in each guideline. Grill et al. [7] reported that different diagnostic tests, pharmacological therapies, or therapeutic maneuvers were used prior to the referral of vertigo patients to a specialist.

One of the reasons for such variation in acute vertigo care might be related to differences in practice between different specialties. In the field of IM, Lerang et al. [8] reported that rheumatologists and non-rheumatologists used different diagnosis and treatment approaches for systemic lupus erythematosus.

There is a possibility that the differences in practice between specialists might result in increased use of resources and cost. However, there is little scientific evidence describing the differences in the type or the quality of acute vertigo care between different specialists. The present study may provide important and new insights to improve acute vertigo care in Japan.

Based on the available literature reports, we hypothesized the presence of significant differences in practice between otolaryngologists and non-otolaryngologists (EM and IM).

## Materials and methods

### Participants

We prospectively collected and analyzed data from the Multicenter Effort to investigate Management of vertigo In Acute Care (MEMAI). The MEMAI study is a multicenter scenario-based survey developed to investigate the variation in acute vertigo care between different specialties, and to standardize vertigo care in Japan. We chose a sample comprising six teaching hospitals (Tokyo Bay Urayasu/Ichikawa Medical Center, Jikei University Hospital, Dokkyo Medical University Saitama Medical Center, Fukui Prefecture Hospital, University of Fukui Hospital, and Tokyo Medical Center) from the Hokuriku and Kanto areas (Fukui, Tokyo, and Saitama) in Japan. All the hospitals were equipped with more than 300 beds.

We included physicians with at least three years of postgraduate year (PGY) experience in departments of otolaryngology, EM, and IM. The exclusion criterion was the non-filling of the questionnaire by the participating physician. We sent a seven-page questionnaire to each research site. Each site investigator was present during the questionnaire administration at each hospital’s educational conference. Prior to questionnaire administration, each site investigator had verbally explained in depth about the study material to the participating physicians. Verbal consent was obtained at the same time. This study was approved by the Ethics Committee of the Tokyo Bay Urayasu/Ichikawa Hospital (approval number: 226).

### Instruments

The study used a questionnaire which consisted of 50 questions and two sections. The questions were prepared by two board-certified EM physicians, two board-certified IM physicians and two board-certified otolaryngologists, and were based on real patient situations.

In the first section, the data collected included gender, post-graduate-year (PGY), specialty, and hospital name. Physicians were also asked about their experience, knowledge, utility, and limitations in imaging modalities used for vertigo care in their hospital.

In the second part of the questionnaire, there were four clinical vignettes of acute vertigo care in the emergency setting. Each case consisted of a patient’s medical history, present complaints, and symptoms. The clinical vignettes included 40 clinical questions regarding diagnosis and treatment of acute vertigo. The final diagnoses of the four vignettes were posterior canal BPPV, vestibular neuritis, Meniere disease, and nonspecific vertigo (Supplemental Appendix 1).

### Diagnosis

Participating physicians read each scenario discussing the four types of vertigo patients without knowing the final diagnosis, and were asked if they would be inclined to perform the Dix-Hallpike test, the head impulse, nystagmus, and test of skew (HINTS), or imaging methods, such as computed tomography (CT) or magnetic resonance imaging (MRI) as central modality, and to mark the possibility of a central cause in each case using a visual analog scale (0 = *no possibility*; 100 = *complete possibility*).

### Treatment

The Japanese clinical review published in the otorhinolaryngological society of Japan recommends prescribing metoclopramide, anti-histamine, and sodium bicarbonate for acute vertigo [9]. Physicians read the scenarios with specific information on final diagnosis and were asked if they would prescribe any or all the following in each case; metoclopramide, antihistamine, sodium bicarbonate, or an Epley maneuver. They were then asked the following question regarding the final disposition: “If the symptom is not diminished after treatment, do you recommend the patient be admitted?”

### Primary data analysis

We used STATA/MP 15.1 (StataCorp LLC) for the data analyses. The data, organized categorically in two groups (otolaryngologist vs. non-otolaryngologist), were compared using the Fisher’s exact test or Mann–Whitney U test. We investigated the associations between physician specialty and diagnostic or treatment option using multiple logistic regression analysis and generalized linear model with a negative binomial distribution and log-link function after covariates. We included variables in the multivariate models based on clinical plausibility and the results of bivariate analysis if an individual p-value was less than 0.25 [10]. A p-value of <0.05 was considered significant.

## Results

### Characteristics of study subjects

During the study period, 151 physicians (84 non-otolaryngologists (48 EM and 36 IM), and 67 otolaryngologists) completed the survey, giving an overall response rate of 98.0%.

Table 1 shows the background of the physicians. In bivariate analysis, gender and PGY were similar between the groups. Otolaryngologists saw significantly more vertigo patients in a month and non-otolaryngologists had significantly more institutional rules to obtain CT scans prior to brain MRI.

**Table 1:** Physician characteristics and knowledge.

Variables	Total (*n* = 151)	Otolaryngologist (*n* = 84)	Non-otolaryngologist (*n* = 64)	p value
PGY, y, Median (IQR)	5.0 (4.0–8.0)	6.0 (4.0–8.0)	5.0 (3.0–9.0)	0.22
Female sex, No. (%)	32 (21.1)	18 (26.9)	14 (16.7)	0.13
Physician’s experience and knowledge about vertigo care				
Number of patients/month, No. (%) by study site				<0.01
1: *0/month*	1 (0.7)	0 (0)	1 (1.2)	
2: *1–5/month*	46 (30.5)	9 (13.4)	37 (44.1)	
3: *6–10/month*	50 (33.1)	15 (22.4)	35 (41.7)	
4: *11–20/month*	22 (14.7)	14 (20.9)	8 (9.5)	
5: *21/months or more*	32 (21.2)	29 (43.28)	3 (3.6)	
Cost of MRI, No. (%) by study site				0.60
1: *5000 yen* ($44)	3 (2.0)	1 (2.8)	0 (0)	
2: *15000 yen* ($133)	80 (53.0)	18 (50)	27 (56.3)	
3: *50000 yen* ($444)	60 (40.0)	15 (41.7)	20 (41.7)	
4: *80000 yen* ($710)	8 (5.3)	2 (5.6)	1 (2.1)	
Detection of CT by study site				0.76
1: *1%*	38 (25.2)	3 (8.3)	16 (33.3)	
2: *2%*	32 (21.2)	10 (27.8)	11 (22.9)	
3: *5%*	44 (29.1)	14 (38.9)	10 (20.8)	
4: *10%*	31 (20.1)	9 (25.0)	8 (16.7)	
5: *30%*	6 (4.0)	0 (0)	3 (6.3)	
Rule to take head CT before brain MRI, No. (%) by study site	20 (13.4)	3 (8.3)	14 (30.4)	<0.01
Limitation in obtaining brain MRI by study site				0.30
1: *Available for 24 hours*	82 (54.3)	17 (47.2)	27 (56.3)	
2: *Some limitation but available for 24 hours*	59 (39.1)	13 (36.1)	19 (39.6)	
3: *No limitation but cannot take MRI at night*	6 (4.0)	2 (5.6)	2 (4.2)	
4: *Only obtain MRI in daytime with some limitation*	4 (2.7)	4 (11.1)	0 (0)	
5: *Without MRI*	0 (0)	0 (0)	0 (0)	

IQR, interquartile range; CT, computed tomography; MRI, magnetic resonance imaging; PGY, post graduate year.

### Bivariate analysis

Tables 2 and 3 show the results of bivariate analysis of vertigo diagnosis and treatment. In the diagnosis section, otolaryngologists preferred significantly fewer head CT scans for the cases of posterior canal BPPV (otolaryngologist (30.0%), non-otolaryngologist (50.6%), p < 0.01) and nonspecific vertigo (otolaryngologist (61.2%), non-otolaryngologist (85.6%), p < 0.01), and were less likely to perform a HINTS procedure for the case of vestibular neuritis (otolaryngologist (62.5%), non-otolaryngologist (84.3%), p < 0.01). Otolaryngologists preferred brain MRI significantly more often for the case of vestibular neuritis (otolaryngologist (41.8%), non-otolaryngologist (17.9%), p < 0.01) and the Dix-Hallpike test for the case of nonspecific vertigo (otolaryngologist (72.7%), non-otolaryngologist (50.6%), p < 0.01). Otolaryngologists were more likely to suspect central causes in the case of posterior canal BPPV (otolaryngologist (8.9%), non-otolaryngologist (8.4%), p < 0.05).

**Table 2:** Results of bivariate analysis in diagnosis by physician type.

	Total (*n* = 151)	Otolaryngologist (*n* = 84)	Non-otolaryngologist (*n* = 64)	p value
BPPV				
Perform head CT, No. (%)	62 (41.3)	20 (30.0)	42 (50.6)	<0.01
Perform brain MRI	26 (17.3)	16 (23.9)	10 (12.1)	0.08
Perform Dix–Hallpike test, No. (%)	142 (94.0)	62 (92.5)	80 (95.2)	0.51
Perform HINTS procedure, No. (%)	105 (72.4)	44 (69.8)	61 (74.4)	0.58
Percentage of central causes, mean. (SD)	10.2 (8.6)	9.0 (8.9)	11.2 (8.4)	<0.05
Vestibular neuritis				
Perform head CT, No. (%)	61 (40.4)	26 (38.8)	35 (41.7)	0.74
Perform brain MRI	43 (28.5)	28 (41.8)	15 (17.9)	<0.01
Perform Dix–Hallpike test, No. (%)	78 (51.7)	35 (52.2)	43 (51.2)	1.00
Perform HINTS procedure, No. (%)	110 (74.8)	40 (62.5)	70 (84.3)	<0.01
Percentage of central causes, mean. (SD)	11.7 (10.1)	12 (8.2)	11.5 (11.4)	0.25
Meniere disease				
Perform head CT, No. (%)	36 (24.0)	15 (22.4)	21 (25.3)	0.71
Perform brain MRI	24 (16.0)	15 (22.4)	9 (10.8)	0.07
Perform Dix–Hallpike test, No. (%)	79 (52.7)	41 (61.2)	38 (45.8)	0.07
Perform HINTS procedure, No. (%)	95 (65.1)	39 (60.9)	56 (68.3)	0.39
Percentage of central causes, mean. (SD)	12.3 (11.7)	13.2 (12.1)	11.5 (11.4)	0.31
Nonspecific vertigo				
Perform head CT, No. (%)	112 (74.7)	41 (61.2)	71 (85.6)	<0.01
Perform brain MRI	96 (64)	44 (65.7)	52 (62.7)	0.74
Perform Dix–Hallpike test, No. (%)	90 (60.4)	48 (72.7)	42 (50.6)	<0.01
Perform HINTS procedure, No. (%)	103 (71.0)	45 (70.3)	58 (71.6)	1.00
Percentage of central causes, mean. (SD)	25.2 (19.2)	25.2 (19.3)	25.2 (19.2)	0.86

BPPV, benign paroxysmal positional vertigo; CT, computed tomography; MRI, magnetic resonance imaging; HINTS the Head Impulse, Nystagmus, Test of Skew procedure.

**Table 3:** Results of bivariate analysis in treatment by physician type.

	Total (*n* = 151)	Otolaryngologist (*n* = 84)	Non-otolaryngologist (*n* = 64)	p value
BPPV				
Prescribe metoclopramide, No. (%)	106 (70.2)	38 (56.7)	68 (81.0)	<0.01
Prescribe anti-histamine, No. (%)	100 (66.2)	42 (62.7)	58 (69.1)	0.41
Prescribe sodium bicarbonate, No. (%)	61 (40.4)	39 (58.2)	22 (26.2)	<0.01
Prescribe Epley maneuver, No. (%)	117 (77.5)	41 (61.2)	76 (90.5)	<0.01
Recommend admitting, No. (%)	81 (54.0)	26 (39.4)	55 (65.5)	<0.01
Vestibular neuritis				
Prescribe metoclopramide, No. (%)	142 (94.0)	59 (88.1)	83 (98.8)	<0.01
Prescribe anti-histamine, No. (%)	116 (76.8)	55 (82.1)	61 (72.6)	0.17
Prescribe sodium bicarbonate, No. (%)	85 (56.3)	61 (91.0)	24 (28.6)	<0.01
Prescribe Epley maneuver, No. (%)	8 (5.3)	1 (1.5)	7 (8,3)	0.08
Recommend admitting, No. (%)	137 (90.7)	66 (98.5)	71 (84.5)	<0.01
Meniere disease				
Prescribe metoclopramide, No. (%)	109 (72.2)	44 (65.7)	65 (77.4)	0.14
Prescribe anti-histamine, No. (%)	113 (74.8)	53 (79.1)	60 (71.4)	0.35
Prescribe sodium bicarbonate, No. (%)	88 (58.3)	61 (91.4)	27 (32.1)	<0.01
Prescribe Epley maneuver, No. (%)	9 (6.0)	3 (4.5)	6 (7.1)	0.73
Recommend admitting, No. (%)	98 (64.9)	44 (65.7)	54 (64.3)	1.00
Nonspecific vertigo				
Prescribe metoclopramide, No. (%)	97 (64.2)	39 (58.2)	58 (69.5)	0.18
Prescribe anti-histamine, No. (%)	98 (64.9)	43 (64.2)	55 (65.5)	1.00
Prescribe sodium bicarbonate, No. (%)	76 (50.3)	51 (76.1)	25 (29.8)	<0.01
Prescribe Epley maneuver, No. (%)	18 (11.9)	4 (6.0)	14 (16.7)	0.04
Recommend admitting, No. (%)	97 (64.2)	37 (55.2)	60 (71.4)	0.04

In the treatment section, otolaryngologists were significantly less likely to use the Epley maneuver to treat posterior canal BPPV (otolaryngologist (61.2%), non-otolaryngologist (90.5%), p < 0.01) or nonspecific vertigo (otolaryngologist (6.0%), non-otolaryngologist (16.7%), p = 0.04). In addition, otolaryngologists had lesser preference for metoclopramide to treat vestibular neuritis (otolaryngologist (88.1%), non-otolaryngologist (98.8%), p < 0.01). Otolaryngologists had a significantly greater preference for sodium bicarbonate to treat posterior canal BPPV (OR, 4.3; 95% CI, 2.11–8.75; p < 0.01), vestibular neuritis (OR, 26.1; 95% CI, 9.81–69.1; p < 0.01), Meniere disease (OR, 21.8; 95% CI, 8.27–57.2; p < 0.01), or nonspecific vertigo (OR, 7.95; 95% CI, 3.76–16.8; p < 0.01).

### Multivariate analysis

Responses to four baseline questions (gender, PGY, the total number of vertigo patients seen in a month, and institutional rules to obtain CT scan prior to brain MRI) in the first section had a p-value <0.25 in bivariate analysis. For the multivariate analysis, we decided to adjust for gender, PGY, and the total number of vertigo patients seen in a month, based on clinical plausibility [10]. There was a large difference in the total number of vertigo patients seen in a month between otolaryngologist and non-otolaryngologist. This may cause different effects on outcomes, we evaluated the interactions of the total number of vertigo patients seen in a month for the relation between outcomes and confounders. However, we did not find any interaction. For the questions regarding imaging modalities such as CT or MRI, we asked the additional question “Do you have an institutional rule that physicians must not take brain MRI without brain CT scan?” After the adjustment, we found that responses to twelve questions (three questions regarding diagnosis, and nine questions regarding treatment) continued to show statistically significant differences (Tables 4 and 5).

**Table 4:** The odds ratio and rate ratio for physician’s willingness to perform each examination and suspicion for central disease among otolaryngologists compared to non-otolaryngologists by multivariate analyses.

	Adjusted OR	95% CI of OR	RR	95% CI of IRR	p value
BPPV					
Perform head CT	0.43	0.19–1.02			0.06
Perform brain MRI	1.61	0.54–4.76			0.39
Perform Dix–Hallpike test	0.27	0.05–1.43			0.13
Perform HINTS procedure	0.71	0.30–1.69			0.44
Percentage of central causes			0.81	0.56–1.18	0.27
Vestibular neuritis					
Perform head CT	0.65	0.28–1.49			0.31
Perform brain MRI	2.01	0.81–4.98			0.13
Perform Dix-Hallpike test	0.84	0.39–1.83			0.67
Perform HINTS procedure	0.16	0.06–0.42			<0.01
Percentage of central causes			1.02	0.69–1.50	0.93
Meniere disease					
Perform head CT	1.00	0.40–2.52			0.99
Perform brain MRI	1.73	0.58–5.22			0.33
Perform Dix-Hallpike test	1.95	0.88–4.31			0.10
Perform HINTS procedure	0.71	0.31–1.62			0.42
Percentage of central causes			1.10	0.75–1.61	0.63
Nonspecific vertigo					
Perform head CT	0.20	0.07–0.56			<0.01
Perform brain MRI	0.73	0.31–1.71			0.47
Perform Dix-Hallpike test	2.48	1.07–5.70			0.03
Perform HINTS procedure	0.67	0.28–1.61			0.37
Percentage of central causes			0.96	0.66–1.40	0.82

OR, odds ratio; IRR, incident, rate ratio.

**Table 5:** The odds ratio and for physician’s willingness to perform each treatment and disposition among otolaryngologists compared to non-otolaryngologists by multivariate analyses.

	Adjusted OR	95%CI of OR	p value
BPPV			
Prescribe metoclopramide, No. (%)	0.26	0.11–0.63	<0.01
Prescribe anti-histamine, No. (%)	0.49	0.21–1.12	0.09
Prescribe sodium bicarbonate, No. (%)	4.08	1.77–9.39	<0.01
Prescribe Epley maneuver, No. (%)	0.17	0.06–0.47	<0.01
Recommend admitting, No. (%)	0.18	0.07–0.45	<0.01
Vestibular neuritis			
Prescribe metoclopramide, No. (%)	0.09	0.01–0.88	0.04
Prescribe anti-histamine, No. (%)	0.96	0.38–2.46	0.95
Prescribe sodium bicarbonate, No. (%)	19.16	6.63–55.26	<0.01
Prescribe Epley maneuver, No. (%)	0.29	0.03–3.14	0.31
Recommend admitting, No. (%)	4.99	0.59–42.29	0.14
Meniere disease			
Prescribe metoclopramide, No. (%)	0.65	0.28–1.54	0.33
Prescribe anti-histamine, No. (%)	0.86	0.35–2.11	0.74
Prescribe sodium bicarbonate, No. (%)	16.24	5.64–46.73	<0.01
Prescribe Epley maneuver, No. (%)	1.12	0.23–5.53	0.89
Recommend admitting, No. (%)	0.75	0.32–1.72	0.49
Nonspecific vertigo			
Prescribe metoclopramide, No. (%)	0.59	0.26–1.33	0.20
Prescribe anti-histamine, No. (%)	0.56	0.24–1.29	0.17
Prescribe sodium bicarbonate, No. (%)	9.61	3.80–24.25	<0.01
Prescribe Epley maneuver, No. (%)	0.47	0.12–1.79	0.27
Recommend admitting, No. (%)	0.27	0.11–0.65	<0.01

In the diagnosis section, otolaryngologists had a significantly lesser preference for head CT in the case of nonspecific vertigo (OR, 0.20; 95% CI, 0.07–0.56; p < 0.01), and for the HINTS procedure in the case of vestibular neuritis (OR, 0.16; 95% CI, 0.06–0.42; p < 0.01). We found no significant difference in the responses to questions regarding the possibility of central causes.

In the treatment section, otolaryngologists had a significantly lesser preference for the Epley maneuver to treat posterior canal BPPV (OR, 0.17; 95% CI, 0.06–0.47; p < 0.01) and for metoclopramide treat posterior canal BPPV (OR, 0.26; 95% CI, 0.11–0.63; p < 0.01) or vestibular neuritis (OR, 0.09; 95% CI, 0.01–0.63; p = 0.04). Otolaryngologists had a significantly greater preference for sodium bicarbonate to treat posterior canal BPPV (OR, 4.08; 95% CI, 1.77–9.39; p < 0.01), vestibular neuritis (OR, 19.16; 95% CI, 6.63–55.26; p < 0.01), Meniere disease (OR, 16.24; 95% CI, 5.64–46.73; p < 0.01), or nonspecific vertigo (OR, 9.61; 95% CI, 3.80–24.25; p < 0.01). Otolaryngologists were less likely to admit posterior canal BPPV patients (OR, 0.18; 95% CI, 0.07–0.45; p < 0.01) or nonspecific vertigo patients (OR, 0.78; 95% CI, 0.11–0.65; p < 0.01).

## Discussion

To the best of our knowledge, the present study is the first to detect differences in acute vertigo care in emergency settings between specialists.

### Diagnosis

In multivariate analysis, even though the use of head CT for the case of nonspecific vertigo was significantly different between otolaryngologists and non-otolaryngologists there was no significant difference in suspicion of central causes based on the history of each scenario; this might be a result of the physicians’ daily bedside practice. In the field of EM, Vanni et al. [11] developed an algorithm to rule out stroke and other life-threatening diseases in acute vertigo for use by emergency physicians. In the field of otolaryngology, Walther [12] reviewed dizziness and vertigo in otolaryngology clinics focusing on peripheral vertigo as well as a multitude of otolaryngology-related diseases involving the inner ear such as barotrauma and fracture of the oto-base; they emphasized the use of more sophisticated technology for accurate diagnoses. It has been reported that unnecessary imaging tests such as head CT and brain MRI were conducted in cases of posterior canal BPPV [13–15]. These findings led us to suspect that unnecessary imaging modalities are done in Japan.

A relatively lesser preference among otolaryngologists for the HINTS procedure in the case of vestibular neuritis was found. The HINTS procedure was found to be more sensitive than early diffusion-weighted MRI for stroke diagnosis [16–19]. Modern vestibular diagnostic tests (eye movement analysis dispose of video documentation systems, etc.) were able to provide objective information [12]. There is possibility that otolaryngologists substitute these tests for the HINTS procedure, and factors such as lack of dissemination of the HINTS procedure and its evidence among otolaryngologists may have influenced this finding. Future studies are needed to explore instituting a training program for the HINTS procedure and to determine whether it decreases use of unnecessary brain MRIs and the relevant costs and time.

Other studies have reported that the cause of the highest number of referrals from the ED to the otolaryngology clinic was peripheral vertigo, and that the most frequent referral diagnosis was nonspecific vertigo [20]. In such cases, otolaryngologists make a specific diagnosis upon examination. It was reported that posterior canal BPPV and vestibular migraine are the most frequently missed etiologies of vertigo, and that the Dix-Hallpike test is the most reliable diagnostic test for posterior canal BPPV [18, 19, 21–23]. Therefore, otolaryngologists may prefer to use the Dix-Hallpike test in cases of nonspecific vertigo to diagnose posterior canal BPPV.

### Treatment

In Japan, there are no differences in physician consultation fee based on the physician specialties, such IM, EM, or otolaryngology [24]. In our questionnaire, the patients with posterior canal BPPV, vestibular neuritis, and Meniere disease complained of nausea. Although metoclopramide is anti-emetic and not an anti-vertigo medication, non-otolaryngologists preferred prescribing metoclopramide in the case of posterior canal BPPV. This might have also been caused by differences in the daily bedside practice. In the ED, ruling out cerebellar bleeding as a cause of vertigo is a high priority task. Non-otolaryngologists tend to control the symptoms of the patients prior to CT in an acute manner. Therefore, non-otolaryngologists might prefer prescribing metoclopramide in cases of posterior canal BPPV or vestibular neuritis causing nausea.

There has been evidence showing that the Epley maneuver was more efficient in treating posterior canal BPPV than placebo and/or drug therapy alone with or without intervention [25, 26]. However, only 60% otolaryngologists were willing to perform Epley maneuver for posterior canal BPPV in our study. We believe it is possible that the Japanese otolaryngologist were less inclined to perform the Epley maneuver because of their overwhelming outpatient volume as well as time burden from the Epley maneuver. However, the Epley maneuver is a well-established and cost-effective treatment method, therefore we should strongly emphasize its effectiveness nationwide. Another possibility is that lack of educational systems which caused lack of knowledge about treatment for posterior canal BPPV. Physicians in otolaryngology clinics should make time for and learn the Epley maneuver to decrease unnecessary admission.

A meta-analysis shows the efficacy for chronic vertigo symptoms and reviews of emergency medicine also recommend using anti-histamine for acute vertigo care [2, 27, 28]. However, only 64.9%– 76.8% otolaryngologists and non-otolaryngologists are willing to prescribe anti-histamine for acute vertigo patients in our study. We need to establish educational systems to disseminate knowledge about efficacy of anti-histamine.

Bivariate analysis showed that at least 25% of the otolaryngologists and non-otolaryngologists were willing to prescribe sodium bicarbonate to treat vertigo patients. Matsunaga et al., and Kawabata et al., [29, 30] in their animal model-based study, observed the efficacy of sodium bicarbonate in vertigo treatment. Aoki [9] commented that the mechanism of sodium bicarbonate action on vertigo was unclear. However, he described two possible mechanisms; dilation of the inner ear’s blood vessels and improvement in acidemia in the inner ear; he surmised that these mechanisms could underlie the treatment of vertigo using sodium bicarbonate. Multivariate analysis revealed that otolaryngologists were significantly more willing to prescribe sodium bicarbonate to treat vertigo patients than non-otolaryngologists. We infer that Japanese otolaryngologists prefer using sodium bicarbonate to treat various types of vertigo even with limited in vitro evidence.

### Limitations

The present study had several limitations. The first was regarding the external validity; even though the study sites were carefully selected to represent geographic diversity, the risk of selection bias should be considered; the sites were all teaching hospitals. Although we found multiple significant differences in acute vertigo care among the specialists, the limited sample size should be considered.

The second limitation was the limited diagnostic skills of the participating physicians. In the diagnosis, to avoid anchoring bias, we intentionally avoided disclosing the final diagnosis in each scenario. Consequently, some of the participating physicians with limited diagnostic skills may not have been able to accurately identify symptom patterns and, thus, the final diagnosis. It is also possible that the participants who read the scenarios in the treatment realized the final diagnosis of each case retrospectively.

The third limitation was that we used questionnaires that were used in a survey investigating theoretical diagnostics and treatments on hypothetical patients. Therefore, the actual practice pattern might have been not reflected.

The forth limitation was multiple comparison. We performed the survey with 50 questions and multiple analyses. We might need to consider that our results could be positive because of multiple comparisons.

The fifth limitation was regarding the potential confounders. We included study variables based on prior knowledge [10]. However, the possibility of unknown confounders not included in this study should be considered.

The sixth limitation was that these results may not be generalizable because this study is based on the Japanese clinical context and the emergency vertigo care in each country is different from that of the Japanese practice.

## Conclusion

There were significant differences in acute vertigo diagnosis and treatment practices between non-otolaryngologists and otolaryngologists from a vignette-based research. These differences might be caused due to variations in the guideline of each specialty. To improve acute vertigo care in Japan, standardized educational systems for acute vertigo are needed.

## Supporting information

S1 Table The survey questions used in the study, in English translation and the original language. (DOCX)
