# Supplementary material for: Comparison of acute vertigo diagnosis and treatment practices between otolaryngologists and non-otolaryngologists: A multicenter scenario-based survey
Source: PLoS One. 2019 Mar 7;14(3):e0213196. doi: 10.1371/journal.pone.0213196 (PMC6405109; doi:10.1371/journal.pone.0213196)
Supplement: S1 Table — (DOCX) [file pone.0213196.s001.docx]

Gender( ),PGY ( ),Department(　　　),Name of hospital( )

Please answer the following question assuming your hospital.

| Question 1：How often do you see patients with dizziness (how many patients) in a month? |
| --- |
| 1. 0 |
| 1. 1-5 |
| 1. 6-10 |
| 1. 11-20 |
| 1. 20< |

| Question 2：Does your hospital have an otorhinolaryngology department? (This question is for those who are not otorhynolaryngologists.) |
| --- |
| Yes　　　　　　　　　　　　　No |

| Question 3：What is the impression of the cost of a simple head MRI? (In the case of 100% out of pocket cost.) |
| --- |
| 1. 5000 yen |
| 1. 15000 yen |
| 1. 50000 yen |
| 1. 80000 yen |

| Question 4：What is the diagnostic rate of head CT on central vertigo? |
| --- |
| 1. 1% |
| 1. 2% |
| 1. 5% |
| 1. 10% |
| 1. 30% |

| Question 5：Are you obligated to take head CT when taking head MRI? |
| --- |
| Yes　　　　　　　　　　　　　No |

| Question 6：In emergency situations, does your hospital take MRI 24 hours a day? |
| --- |
| 1. We can take MRI 24 hours a day. |
| 1. We can take MRI 24 hours a day under certain conditions. |
| 1. We can take MRI only during the day, but not at night. |
| 1. We can take MRI only during the day under certain conditions, but not at night. |
| 1. No MRI |

*Patient with vertigo

Please answer the following questions assuming that you work at a general hospital with internal medicine, emergency and otorhinolaryngology departments. There are sufficient number of hospital beds available and good access to CT and MRI. A patient visited during day time on a holiday.

| Case A：  Patient is a 60-year-old male, who has a history of hypertension. He had vertigo when waking up in the morning on that day. He does not have symptoms while resting, but vertigo occurs upon body movement, and disappears in about one minute. He does not have tinnitus or hearing difficulty. He can maintain a standing position. |
| --- |
| 1. Do you order head CT? |
| Yes　　　　　　　　　　　　　No |
| 1. Do you order brain MRI? |
| Yes　　　　　　　　　　　　　No |
| 1. Do you perform Dix-Hallpike maneuver? |
| Yes　　　　　　　　　　　　　No |
| 1. Do you perform HINTS method? |
| Yes　　　　　　　　　　　　　No |
| 1. What % do you think central vertigo is hidden in this case? |
| 0 10 20 30 40 50 60 70 80 90 100  \|______\|______\|______\|______\|______\|______\|______\|______\|______\|______\| |

| Case B：  A female in the 30’s with no particular history, who had cold symptoms a week ago. Patient started to have tinnitus one hour prior to her visit, and then high-grade vertigo appeared. She has had vomiting. She does not complain tinnitus or difficulty of hearing. There is no neurological abnormality, but a gaze nystagmus test showed nystagmus on the left horizontal direction.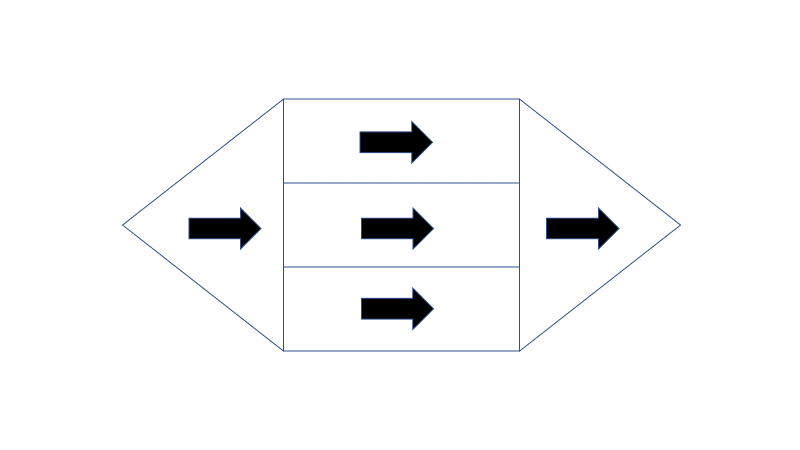 |
| --- |
| 1. Do you order head CT? |
| Yes　　　　　　　　　　　　　No |
| 1. Do you order brain MRI? |
| Yes　　　　　　　　　　　　　No |
| 1. Do you perform Dix-Hallpike maneuver? |
| Yes　　　　　　　　　　　　　No |
| 1. Do you perform HINTS method? |
| Yes　　　　　　　　　　　　　No |
| 1. What % do you think central vertigo is hidden in this case? |
| 0 10 20 30 40 50 60 70 80 90 100  \|______\|______\|______\|______\|______\|______\|______\|______\|______\|______\| |

| Case C：  A male in the 40’s who has been diagnosed with Meniere’s disease has visited the emergency room for severe vertigo several times a year. He visited the emergency room with a similar case of vertigo and vomiting, which made body movement difficult. Patient complains about difficulty of hearing in the right ear. He can maintain a standing position. |
| --- |
| 1. Do you order head CT? |
| Yes　　　　　　　　　　　　　No |
| 1. Do you order brain MRI? |
| Yes　　　　　　　　　　　　　No |
| 1. Do you perform Dix-Hallpike maneuver? |
| Yes　　　　　　　　　　　　　No |
| 1. Do you perform HINTS method? |
| Yes　　　　　　　　　　　　　No |
| 1. What % do you think central vertigo is hidden in this case? |
| 0 10 20 30 40 50 60 70 80 90 100  \|______\|______\|______\|______\|______\|______\|______\|______\|______\|______\| |

| Case D：  A male in the 50’s, who has a history of hypertension but no medication history, visited an emergency outpatient for floating dizziness that had lasted for three days. The symptom continues throughout the day without any exacerbation or remission factor. Patient does not complain about tinnitus or hearing difficulty and can maintain a standing position. Physical examination including vital signs and neurological examination showed no abnormality. |
| --- |
| 1. Do you order head CT? |
| Yes　　　　　　　　　　　　　No |
| 1. Do you order brain MRI? |
| Yes　　　　　　　　　　　　　No |
| 1. Do you perform Dix-Hallpike maneuver? |
| Yes　　　　　　　　　　　　　No |
| 1. Do you perform HINTS method? |
| Yes　　　　　　　　　　　　　No |
| 1. What % do you think central vertigo is hidden in this case? |
| 0 10 20 30 40 50 60 70 80 90 100  \|______\|______\|______\|______\|______\|______\|______\|______\|______\|______\| |

*Patient with vertigo

Please answer the following questions assuming that you work at a general hospital with internal medicine, emergency and otorhinolaryngology departments. There are sufficient number of hospital beds available and good access to CT and MRI. A patient visited during day time on a holiday.

| Case A：BPPV  Patient is a 60-year-old male, who has a history of hypertension. He had vertigo when waking up in the morning on that day. He does not have symptoms while resting, but vertigo occurs upon body movement, and disappears in about one minute. He does not have tinnitus or hearing difficulty. He can maintain a standing position. Dix-Hallpike test (positional nystagmus test) was performed, which provoked vertigo at right 45 degrees head-hanging position. Rotatory nystagmus toward the right (counterclockwise viewed from the operator) disappeared within a minute. When returning to the sitting position, nystagmus toward the opposite direction is provoked.  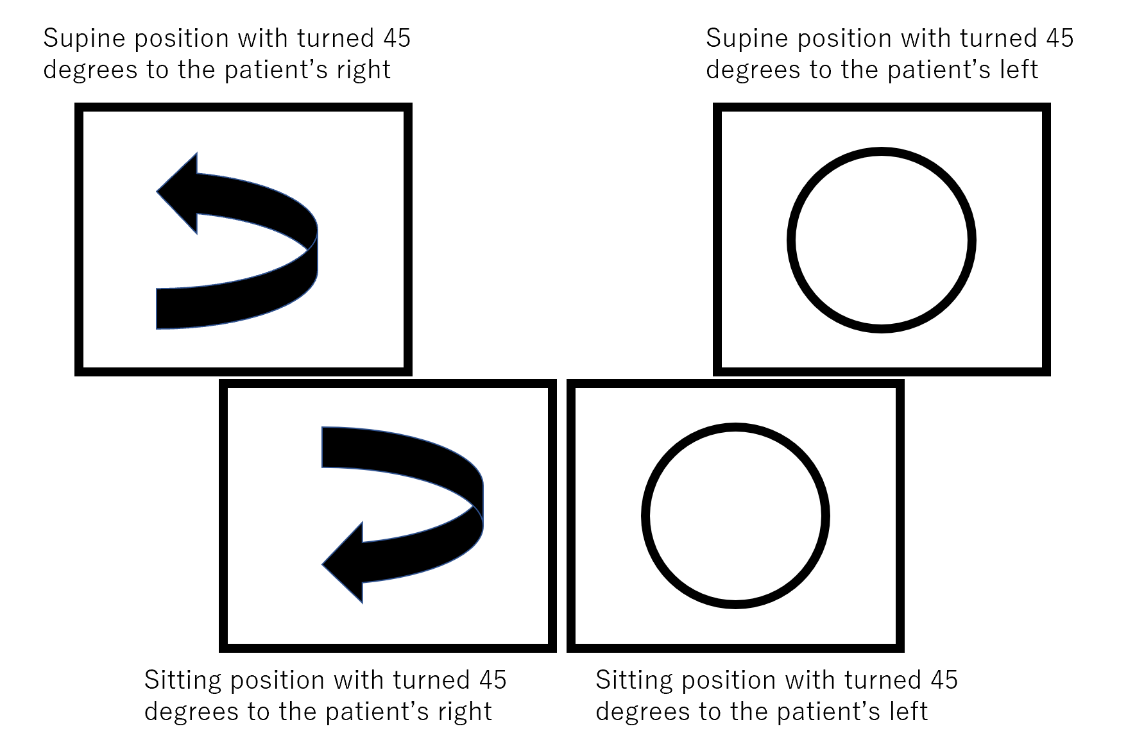  Based on the physical findings, BPPV is suspected. |
| --- |
| 1. Do you prescribe metoclopramide? |
| Yes　　　　　　　　　　　　　No |
| 1. Do you prescribe antihistamine? |
| Yes　　　　　　　　　　　　　No |
| 1. Do you prescribe sodium bicarbonate? |
| Yes　　　　　　　　　　　　　No |
| 1. Do you perform Epley maneuver? |
| Yes　　　　　　　　　　　　　No |
| 1. If the symptom is not diminished after treatment, do you recommend the patient be admitted? |
| Yes　　　　　　　　　　　　　No |

| Case B：Vestibular neuritis  A female in the 30’s with no particular history, who had cold symptoms a week ago. Patient started to have tinnitus one hour prior to her visit, and then high-grade vertigo appeared. She has had vomiting. She does not complain tinnitus or difficulty of hearing. There is no neurological abnormality, but a gaze nystagmus test showed nystagmus on the left horizontal direction. Based on the physical findings, vestibular neuritis is suspected.  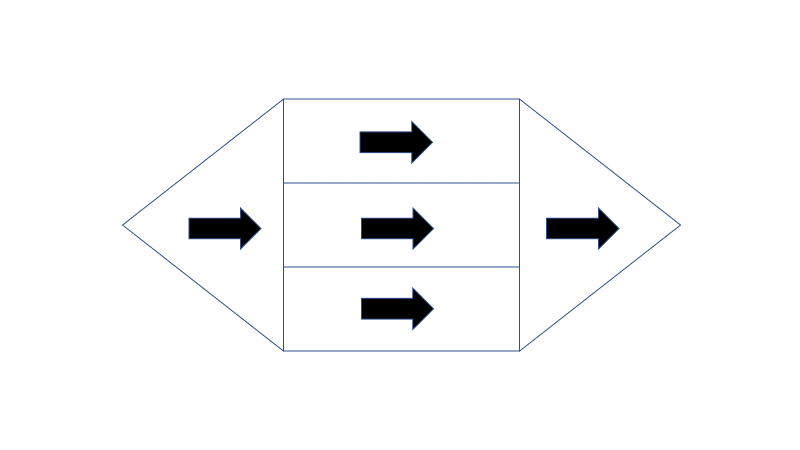 |
| --- |
| 1. Do you prescribe metoclopramide? |
| Yes　　　　　　　　　　　　　No |
| 1. Do you prescribe antihistamine? |
| Yes　　　　　　　　　　　　　No |
| 1. Do you prescribe sodium bicarbonate？ |
| Yes　　　　　　　　　　　　　No |
| 1. Do you perform Epley maneuver？ |
| Yes　　　　　　　　　　　　　No |
| 1. If the symptom is not diminished after treatment, do you recommend the patient be admitted? |
| Yes　　　　　　　　　　　　　No |

| Case C：Meniere disease  A male in the 40’s who has been diagnosed with Meniere’s disease has visited the emergency room for severe vertigo several times a year. He visited the emergency room with a similar case of vertigo and vomiting, which made body movement difficult. Patient complains about difficulty of hearing in the right ear. He can maintain a standing position. Based on the physical findings, Meniere disease is suspected. |
| --- |
| 1. Do you prescribe metoclopramide? |
| Yes　　　　　　　　　　　　　No |
| 1. Do you prescribe antihistamine? |
| Yes　　　　　　　　　　　　　No |
| 1. Do you prescribe sodium bicarbonate? |
| Yes　　　　　　　　　　　　　No |
| 1. Do you perform Epley maneuver？ |
| Yes　　　　　　　　　　　　　No |
| 1. If the symptom is not diminished after treatment, do you recommend the patient be admitted? |
| Yes　　　　　　　　　　　　　No |

| Case D：Nonspecific vertigo  A male in the 50’s, who has a history of hypertension but no medication history, visited an emergency outpatient for floating dizziness that had lasted for three days. The symptom continues throughout the day without any exacerbation or remission factor. Patient does not complain about tinnitus or hearing difficulty and can maintain a standing position. Physical examination including vital signs and neurological examination showed no abnormality.  Brain MRI shows no abnormality. Based on the physical findings, nonspecific vertigo is suspected. |
| --- |
| 1. Do you prescribe metoclopramide? |
| Yes　　　　　　　　　　　　　No |
| 1. Do you prescribe antihistamine? |
| Yes　　　　　　　　　　　　　No |
| 1. Do you prescribe sodium bicarbonate? |
| Yes　　　　　　　　　　　　　No |
| 1. Do you perform Epley maneuver？ |
| Yes　　　　　　　　　　　　　No |
| 1. If the symptom is not diminished after treatment, do you recommend the patient be admitted? |
| Yes　　　　　　　　　　　　　No |

性別（　　）、卒後年数（　　）年、診療科（　　　）科、施設名

あなたの働いている病院を想定して質問に答えてください。

| 質問1：月にめまいを診る頻度（人） |
| --- |
| 1. 0 |
| 1. 1-5 |
| 1. 6-10 |
| 1. 11-20 |
| 1. 20< |

| 質問2：あなたの病院は耳鼻科がありますか？（耳鼻科医以外の方が対象） |
| --- |
| はい　　　　　　　　　　　　　　　　　　　　　いいえ |

| 質問3：頭部単純MRIの1回の値段の印象(10割負担の場合で) |
| --- |
| 1. 5000円 |
| 1. 15000円 |
| 1. 50000円 |
| 1. 80000円 |

| 質問4：頭部CTの中枢性めまいに対する診断率 |
| --- |
| 1. 1% |
| 1. 2% |
| 1. 5% |
| 1. 10% |
| 1. 30% |

| 質問5：あなたの病院は頭部MRIを取る際に頭部CTを取ることが義務づけられていますか？ |
| --- |
| はい　　　　　　　　　　　　　　　　　　　　　いいえ |

| 質問6：あなたの施設は緊急でMRIを24時間緊急でとれますか？ |
| --- |
| 1. 24時間緊急で撮影できる |
| 1. 条件付きで24時間撮影できる |
| 1. 夜間不可、日中のみ撮影できる |
| 1. 夜間不可、日中のみ条件付きで撮影できる |
| 1. MRIがない |

*めまいの患者

あなたは内科、救急科、耳鼻科がある総合病院で働いています。入院ベッドは十分にあり、CT、MRIへのアクセスは非常によいです。患者が受診したのは休日の日勤帯です。これらを想定して質問にこたえてください。

| 症例A：  60歳男性、高血圧の既往のある患者。来院同日起床時に回転性めまいを自覚。安静にしていると症状がでないが体動でめまいが出現し1分程度で消失する。耳鳴り、耳の聞こえにくさの訴えはない。立位の保持は可能。 |
| --- |
| 1. CTを撮りますか？ |
| 賛成する　　　　　　　　　　　　　反対する |
| 1. MRIを撮影しますか？ |
| 賛成する　　　　　　　　　　　　　反対する |
| 1. Dix-Hallpike試験（頭位変換眼振検査）を行いますか？ |
| 賛成する　　　　　　　　　　　　　反対する |
| 1. HINTS methodを行いますか？ |
| 賛成する　　　　　　　　　　　　　反対する |
| 1. この症例で中枢性めまいが隠れている頻度は何％くらい？ |
| 0 10 20 30 40 50 60 70 80 90 100  \|______\|______\|______\|______\|______\|______\|______\|______\|______\|______\| |

| 症例B：  30代女性、特に既往なし。1週間前に感冒症状があった。来院1時間前より、耳鳴りが出現しその後高度のめまいが出現した。嘔吐を伴う。患者より耳鳴り、耳の聞こえにくさの訴えはない。神経学的異常はなく、注視眼振検査にて左水平方向性の眼振があります。  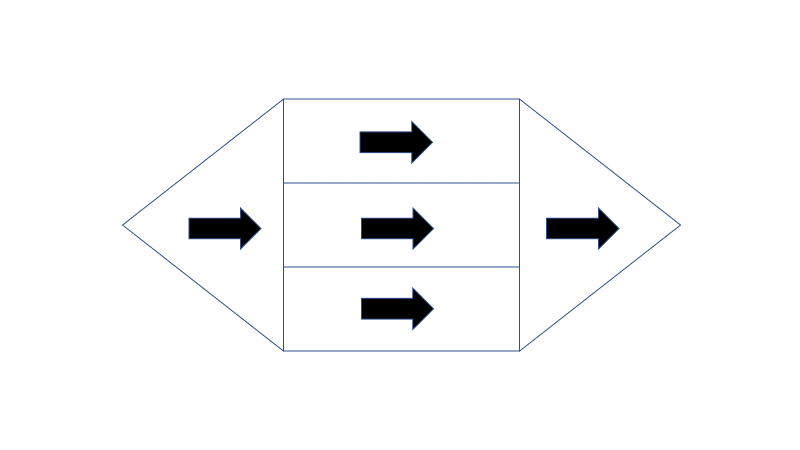 |
| --- |
| 1. CTを撮りますか？ |
| 賛成する　　　　　　　　　　　　　反対する |
| 1. MRIを撮影しますか？ |
| 賛成する　　　　　　　　　　　　　反対する |
| 1. Dix-Hallpike試験（頭位変換眼振検査）を行いますか？ |
| 賛成する　　　　　　　　　　　　　反対する |
| 1. HINTS methodを行いますか？ |
| 賛成する　　　　　　　　　　　　　反対する |
| 1. この症例で中枢性めまいが隠れている頻度は何％くらい？ |
| 0 10 20 30 40 50 60 70 80 90 100  \|______\|______\|______\|______\|______\|______\|______\|______\|______\|______\| |

| 症例C：  40代男性、すでにメニエール病と診断され年に数回強いめまいが出現し救急外来を受診している。本日も同様のめまい、嘔気が出現し体動困難なため救急受診した。右の耳の聞こえにくさを訴えている。立位の保持は可能。 |
| --- |
| 1. CTを撮りますか？ |
| 賛成する　　　　　　　　　　　　　反対する |
| 1. MRIを撮影しますか？ |
| 賛成する　　　　　　　　　　　　　反対する |
| 1. Dix-Hallpike試験（頭位変換眼振検査）を行いますか？ |
| 賛成する　　　　　　　　　　　　　反対する |
| 1. HINTS methodを行いますか？ |
| 賛成する　　　　　　　　　　　　　反対する |
| 1. この症例で中枢性めまいが隠れている頻度は何％くらい？ |
| 0 10 20 30 40 50 60 70 80 90 100  \|______\|______\|______\|______\|______\|______\|______\|______\|______\|______\| |

| 症例D：  50歳男性、高血圧の既往あり、薬剤歴なし。3日間続く浮動性めまいで救急外来を受診。増悪緩解因子なく、一日中続いている。耳鳴り、耳の聞こえにくさの訴えはない。立位の保持は可能。バイタルサイン、神経診察を含め身体診察上、異常なし。 |
| --- |
| 1. CTを撮りますか？ |
| 賛成する　　　　　　　　　　　　　反対する |
| 1. MRIを撮影しますか？ |
| 賛成する　　　　　　　　　　　　　反対する |
| 1. Dix-Hallpike試験（頭位変換眼振検査）を行いますか？ |
| 賛成する　　　　　　　　　　　　　反対する |
| 1. HINTS methodを行いますか？ |
| 賛成する　　　　　　　　　　　　　反対する |
| 1. この症例で中枢性めまいが隠れている頻度は何％くらい？ |
| 0 10 20 30 40 50 60 70 80 90 100  \|______\|______\|______\|______\|______\|______\|______\|______\|______\|______\| |

*めまいの患者

あなたは内科、救急科、耳鼻科がある総合病院で働いています。入院ベッドは十分にあり、CT、MRIへのアクセスは非常によいです。患者が受診したのは休日の日勤帯です。これらを想定して質問にこたえてください。

| 症例A：BPPV  60歳男性、高血圧の既往のある患者。来院同日起床時に回転性めまいを自覚。安静にしていると症状がでないが体動でめまいが出現し1分程度で消失する。耳鳴り、耳の聞こえにくさの訴えはない。立位の保持は可能。Dix-Hallpike試験（頭位変換眼振検査）を行い、右45度懸垂頭位でめまいが出現した。眼振は右回り（験者からみて反時計回り）で1分以内に消失した。座位に戻すと反対方向性の眼振が出現する。  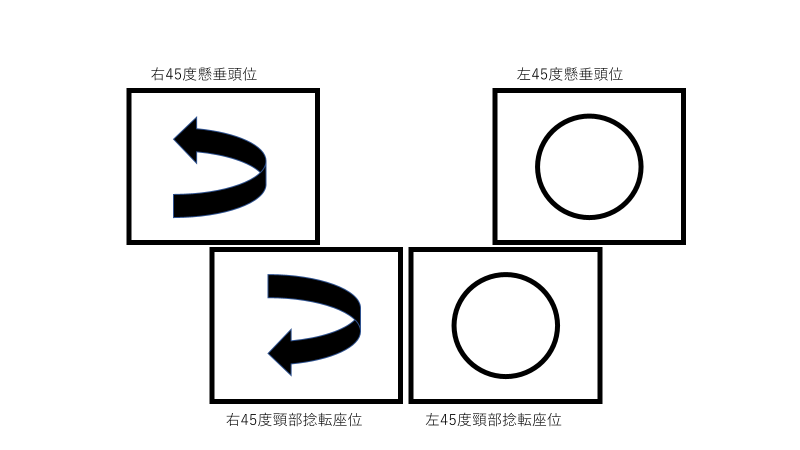  身体所見上BPPVが疑われます。 |
| --- |
| 1. メトクロプラミド（プリンペラン）を投与しますか？ |
| 賛成する　　　　　　　　　　　　　反対する |
| 1. 抗ヒスタミン薬を投与しますか？ |
| 賛成する　　　　　　　　　　　　　反対する |
| 1. 重炭酸（メイロン）を投与しますか？ |
| 賛成する　　　　　　　　　　　　　反対する |
| 1. Epley法を行いますか？ |
| 賛成する　　　　　　　　　　　　　反対する |
| 1. 治療後、めまい症状は残存しています。入院させますか？ |
| 賛成する　　　　　　　　　　　　　反対する |

| 症例B：前庭神経炎  30代女性、特に既往なし。1週間前に感冒症状があった。来院1時間前より、耳鳴りが出現しその後高度のめまいが出現した、嘔吐が伴い、目が開けていられない。患者本人より耳鳴り、耳の聞こえにくさの訴えはない。神経学的異常はなく、注視眼振検査にて左水平方向性の眼振があります。病歴からは前庭神経炎が疑われます。  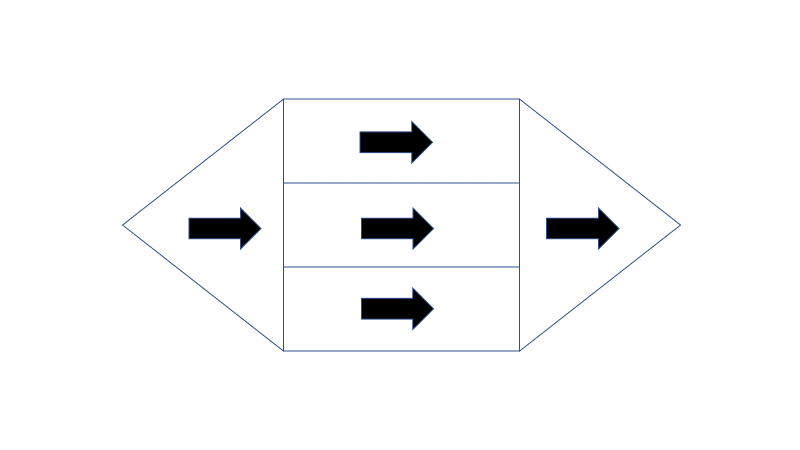 |
| --- |
| 1. メトクロプラミド（プリンペラン）を投与しますか？ |
| 賛成する　　　　　　　　　　　　　反対する |
| 1. 抗ヒスタミン薬を投与しますか？ |
| 賛成する　　　　　　　　　　　　　反対する |
| 1. 重炭酸（メイロン）を投与しますか？ |
| 賛成する　　　　　　　　　　　　　反対する |
| 1. Epley法を行いますか？ |
| 賛成する　　　　　　　　　　　　　反対する |
| 1. 治療後、めまい症状は残存しています。入院させますか？ |
| 賛成する　　　　　　　　　　　　　反対する |

| 症例C：メニエール病  40代男性、すでにメニエール病と診断され年に数回強いめまいが出現し救急外来を受診している。本日も同様のめまい、嘔気が出現し体動困難なため救急受診した。右の耳の聞こえにくさを訴えている。立位の保持は可能。メニエール病が疑われます。 |
| --- |
| 1. メトクロプラミド（プリンペラン）を投与しますか？ |
| 賛成する　　　　　　　　　　　　　反対する |
| 1. 抗ヒスタミン薬を投与しますか？ |
| 賛成する　　　　　　　　　　　　　反対する |
| 1. 重炭酸（メイロン）を投与しますか？ |
| 賛成する　　　　　　　　　　　　　反対する |
| 1. Epley法を行いますか？ |
| 賛成する　　　　　　　　　　　　　反対する |
| 1. 治療後、めまい症状は残存しています。入院させますか？ |
| 賛成する　　　　　　　　　　　　　反対する |

| 症例D：特定不能な末梢性めまい  50歳男性、高血圧の既往あり、薬剤歴なし。3日間続く浮動性めまいで救急外来を受診。増悪緩解因子なく、一日中続いている。バイタルサイン、神経診察を含め身体診察上、異常なし。耳鳴り、耳の聞こえにくさの訴えはない。立位の保持は可能。  MRIで異常なく、中枢性のめまいは疑わないが何らかの末梢性めまいを疑います。 |
| --- |
| 1. メトクロプラミド（プリンペラン）を投与しますか？ |
| 賛成する　　　　　　　　　　　　　反対する |
| 1. 抗ヒスタミン薬を投与しますか？ |
| 賛成する　　　　　　　　　　　　　反対する |
| 1. 重炭酸（メイロン）を投与しますか？ |
| 賛成する　　　　　　　　　　　　　反対する |
| 1. Epley法を行いますか？ |
| 賛成する　　　　　　　　　　　　　反対する |
| 1. 治療後、めまい症状は残存しています。入院させますか？ |
| 賛成する　　　　　　　　　　　　　反対する |

**S1 Table. The survey questions used in the study, in English translation and the original language.**
